# Baltimore Medical Association

**Published:** 1880-07

**Authors:** 


### BALTIMORE MEDICAL ASSOCIATION.

*Meeting Held April 26th*, 1880.

John F. Monmonier, M.D., President, in the chair, E. F. Cordell, M.D., Recording Secretary.

*Apoplexy.*—Dr. Taneyhill reported a case of apoplexy in a man aged eighty-five. He had been shingling a roof when experiencing a sensation of giddiness, he descended to the ground, where he was found by Dr. T. speechless, with sterorous breathing and right hemiplegia. An enema was ordered, followed by a dose of magnesium sulphate. On the following day he was partially conscious, but unable to protrude his tongue. He was now put upon iodide potassium, gr. xx, every four hours. On the third day he could raise his right arm; his tongue diverged toward the left. On the fifth day he began to articulate, but unintelligibly. On the 6th day (yesterday) he could elevate his arm, and assist in dressing himself. To-day he uses his knife in eating. There was no aphasia; the urine was not examined.

*Jamaica Dogwood.*—Dr. Kemp reported the trial of this remedy in three cases. In a case of diarrhœa, accompanied by pain, tormina and tenemus, these symptoms were relieved by ʒss, and constipation produced. He administered it to a young woman suffering from dysmenorrhœa; her pains were relieved as much as by the ordinary remedies. The third case was a most unfavorable one; the patient suffering from softening of the brain, requiring to be constantly controlled, and needing as much as two grains of morphia to keep him quiet; favorable results were hardly to be expected here, yet ʒi ss seemed to exert some calming effect. No change of pulse was observed. It produced decided constipation. On the whole, to judge by so small an experience, its effects were quite as favorable as were claimed for it.

*Cascara Cordial*—Dr. Morris had made trial of this remedy and had found it a very agreeable laxative. Dr. Ashby said the cascara sagrada had been employed successfully for the same effect at the University Hospital. The bitter taste may be relieved by adding syrup of ginger.

*Urethral Stricture from Eating Asparagus.*—Dr. Maddux reported the case of a gentleman who suffered from retention of urine, necessitating the repeated introduction of the catheter. He had indulged very freely in asparagus, and the urine drawn exhaled the characteristic odor very strongly. The stricture was situated near the neck of the bladder. A No. 8 catheter was passed, but was very tight; on remaining in five or ten minutes, it became loose. The patient was of intemperate habits.

*Pain and the Therapeutic Value of Medicines for its Relief.*—This was the regular subject for discussion, which was introduced by Dr. C. H. Jones, with a paper.

Dr. Erich objected to the opinion that opium must not be used during the first twelve months of life. Infants suffer as much as adults, and pain alone is capable of causing death, of which fact he had seen many instances. He noted the omission of salicylic acid in the paper, a remedy which produces relief more promptly in acute rheumatism than any other, and in neuralgias, exerts often an equally remarkable influence, which is then apt to be permanent. The effect of bromide of potassium in uterine disease also deserved mention; in one case under his observation the relief was very remarkable. The patient suffered from congenital absence of the vagina, in consequence of which the uterus was rapidly increasing in size, forming a large abdominal tumor. Severe pains were experienced, requiring large doses of morphia An attempt at the formation of an artificial vagina had been made previously to her coming under his care, but abandoned. The patient was well developed otherwise; no vagina could be felt through the rectum, but only the recto-vesical septum and the distended uterus. The patient would not consent to further operative interference, but demanded palliative treatment; bromide of potassium was, therefore, ordered in ℨss. doses, three times daily, for one week before menstrual period. The result was so great a relief that morphia was no longer required during the periods; further enlargement was prevented, and the tumor diminished somewhat in size. The patient enjoys very good health, only complaining of the languor and depression resulting from the artificial anæmia produced by the bromide, and for which strychnia, iron and quinine are given for one week after each period. To prevent the eruption due to the same agent Dr. Erich uses Fowler's solution. Dr. Uhler referred to the tincture of gelseminum as generally reliable in relieving facial neuralgia, used with or without opium.

Dr. Arnold quoted the saying of Virchow, that therapeutics is the lumber-room of medicine. It is certainly the most backward of all the departments of our science. The final appeal in question of therapeutics is, after all, personal experience at the bedside, and how shall we guage this personal experience? One should think that, at least in the matter of pain, there should be no division of opinion respecting the efficacy of certain remedial agencies. But pain is the subjective symptom *par excellence*, and for this very reason opens a wide field for discrepancies respecting the effects of the various sedatives at our disposal. Believing implicitly in the efficiency and promptness of the action of opium, or its preparations, he cannot conscientiously resort to other and less tried medicinal substances for the relief of pain.

Dr. Williams referred to the alleged anodyne effects of chloral, of which he expressed doubt; it puts the patient to sleep in spite of the pain, but on awaking, the pain returns. In obstetrical practice nothing relieves pain and relaxes the os uteri so well as chloral; an injection into the rectum of 30 to 40 grains in warm milk often enables the accoucheur to go away safely for five or six hours, and attend to other business. It does not interfere with the uterine contractions which go on the same as though it were not given; the only objection to it is the prolonged effect. As for opium, nothing can equal it in anodyne effect, but the terrible nausea it often causes is a very great objection to its use. Gelseminum will relieve supra-orbital neuralgia without causing subsequent nausea. In peritonitis nothing equals opium. Many inflammatory diseases may be aborted by a full dose of the same. In some patients the nausea may be relieved to a great extent by combining it with belladonna; this practice is commendable in hypodermic use, because we cannot predicate the effect of opium in a stranger. The combination is both philosophical and efficient.

Dr. Erich objected to chloral in the latter stages of labor, because it is necessary to give very large doses, and thereby stupefy the patient. He prefers chloroform, using it, however, only when the pains are present. There are different sorts of nausea following the use of opium; when it comes on five or six hours after the administration, it may be controlled by keeping the patient's head down, and putting nothing into the stomach. He follows this rule himself in using the drug for the use of sick headaches, tapering off with gradually diminishing doses; for instance, taking half a grain at the beginning of the attack, quarter of a grain four hours after, and an eighth ot a grain after a similar interval of time, also cautiously resuming food. The recumbency should be continued twelve to fifteen hours. Thirst is not experienced if vomiting be avoided. In treating colic, his plan is to administer a hypodermic injection of atropia, and at the same time a full dose of calomel, the latter produces operations after some hours, and is not objectionable given simultaneously with the atropia.

Dr. Kemp said relief from the nausea could be obtained by the use of citrate of caffein.

Dr. Arnold thought the importance of this nausea was much exaggerated; a very small percentage of persons suffer from it; it is soon over, and in the relief obtained from severe suffering, it is forgotten. A little thein or caffein may be used as a palliative, if desired. In severe pain opium is indicated always and under all circumstances.

Dr. Gibbons said that a friend of his, a druggist, having occasion to employ opium found that by snuffing it up the nose he obtained the anodyne effect without the nausea—half grain was ordinarily required.

*The Cause of Disease.*—Dr. J. T. Smith read a paper upon this subject.

The association then adjourned.

